# Miniaturized Sensors Registering the Long-Term Course of Suture Tension In Vivo under Varying Intra-Abdominal Pressure

**DOI:** 10.3390/s18061729

**Published:** 2018-05-28

**Authors:** Jörg Höer, Oliver Wetter

**Affiliations:** 1 Hochtaunuskliniken Bad Homburg, Department of General and Visceral Surgery, Zeppelinstrasse 20, D-61352 Bad Homburg, Germany; 2 Fachhochschule Bielefeld, Campus Minden, Fachbereich Technik, Artilleriestrasse 9, D-32427 Minden, Germany; oliver.wetter@fh-bielefeld.de

**Keywords:** suture tension, incisional hernia, laparotomy closure, laparotomy, implantable sensor, tactile control, surgical complications, surgical technique

## Abstract

Background: Failure of laparotomy closure develops after up to 20% of abdominal operations. Suture tension has an influence on the quality of tissue regeneration. No sensors are available to register suture tension dynamics in vivo. Methods: In a series of animal experiments, the effect of suture tension on the ultrastructure of the healing incision was examined. Surgeons’ ability to suture with target tension was tested. An implantable sensor and data logger were developed and tested experimentally in sutures closing midline laparotomies in pigs both under normal and elevated intra-abdominal pressure. Results: High suture tension has a negative influence on the regeneration of laparotomy incisions. Running sutures for laparotomy closure lose 45% of their initial tension over periods of 23 h. Intermittent elevation of intra-abdominal pressure to 30 mm Hg leads to a near total loss of suture tension after 23 h. Conclusion: Surgeons are not able to control and reproduce suture tension. Suture tension dynamics can be measured in vivo by the sensor developed. Further research is needed to define a tissue-specific suture tension optimum to reduce the incidence of complications after laparotomy. Techniques for laparotomy closure need to be modified.

## 1. Introduction

In the year 2000, van Geldere published an article entitled “One hundred years of abdominal wound dehiscence and nothing has changed” [1]. This is the factual summary of an objective analysis of surgical reality. 

Closing abdominal incisions is an everyday surgical routine, but acute and chronic failure of laparotomy closure with the development of either ruptured abdomen or incisional hernia (IH) remain at high levels between 12 and 20% and are the postoperative complications most often requiring surgical re-intervention. In 2012, about 190.000 IHs were repaired in in-patients in the United States at costs of $3.2 billion US [2].

For decades, surgical research focused on suture material, suture technique, and risk-factor analysis to improve the results of primary abdominal closure. This research had no relevant effect on IH incidence.

In 2010, Diener et al. analyzed five systematic reviews and 14 trials including 7711 patients to evaluate the optimal technique and material for abdominal fascia closure after midline laparotomy. The authors concluded that no further trials for an evaluation of technique and available materials for elective abdominal fascial closure should be conducted [3]. In 2015, the ongoing research to ameliorate the results of laparotomy closure has been condensed in the European Hernia Society (EHS) guidelines on the closure of abdominal wall incisions [4]. The EHS recommended

- a non-midline approach to the abdominal cavity whenever feasible,
- a continuous suturing technique,
- slowly absorbable suture material, and
- small-bite suture technique with a suture-length–wound-length ratio of at least 4:1.

Although research has proven that suture tension has a relevant influence on the quality of wound healing, a possible influence of suture tension on fascial healing is not mentioned in these guidelines and no proposition has been made regarding the optimal suture tension to precondition undisturbed fascial healing.

The aim of measuring suture tension and suture tension dynamics has led to the development of sensor devices by different scientific working groups. The devices described in the literature are mostly bulky, they only allow for the measurement of suture tension between stitches and not along a suture line, and they are not able to register suture tension over a relevant period of time after completion of the suture (see Section 4.1).

As a consequence, we have developed a sensor-based “knot-trainer” to investigate the reproducibility and precision of surgical knotting [5] and an implantable sensor device capable of long-term suture tension measurement in vivo after completion of laparotomy closure. The developmental work and sensor realization has already been described in [6].

### Study Design and Aims

This study compares suture tension dynamics in laparotomy incisions in pigs measured by an implantable sensor device under the influence of different intra-abdominal pressure levels. Together with the results obtained from the experimental use of the “knot-trainer,” it aims at defining a tissue-specific suture tension optimum avoiding tissue damage by inadequate suture tension and at triggering research for alternative techniques for laparotomy closure.

## 2. Material and Methods

### 2.1. A Sensor Device to Measure Suture Tension Reproducibility and Precision In Vitro

**Figure 1:**
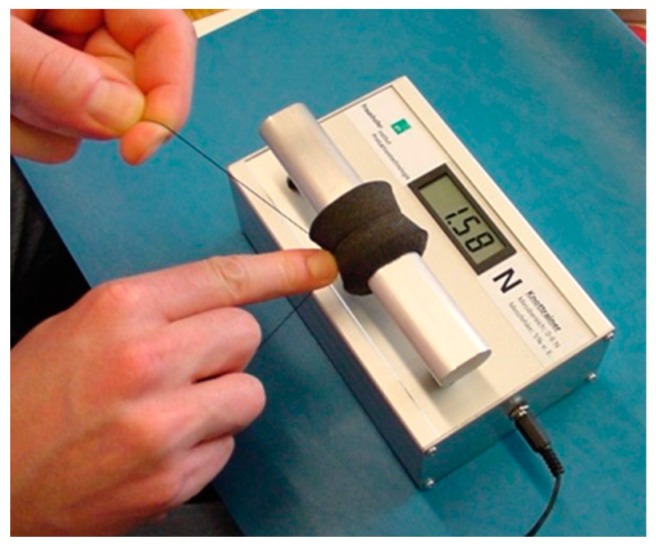
Knot-trainer with display visible [6].

Together with the Fraunhofer Institute for Production Technology (Aachen, Germany) we developed a “knot-trainer” allowing the measurement of simulated fascial sutures. A capacitive resistor (0.2 mm × 4 mm × 0.1 mm) embedded in fluid silicone was mounted on an aluminum bar and covered with polyurethane-foam of the same elasticity and frictional resistance like the abdominal components enclosed in fascial sutures. Suture tension measured in N was displayed digitally, the display remaining invisible to the tested person during the experiment (Figure 1).

Each of 17 abdominal surgeons had to knot 21 single sutures over the sensor, 7 knots with a suture tension they considered as “low” for fascial closure, 7 knots with a tension they considered as “adequate,” and 7 sutures with “high” suture tension. Mean values, interquartile ranges, and reproducibility/precision were analyzed (Table 1).

### 2.2. Experimental Evaluation of Suture Tension Using a Macro-Sensor

To acquire information about the suture tension and cutting forces of the thread that have to be expected during laparotomy closure a pilot test was performed in pigs. In cooperation with the Fraunhofer Institute for Production Technology (Aachen, Germany), a load cell measuring 60 mm × 40 mm × 24 mm was fixed on a mount lateral to the operating field during fascial closure. A specially designed clamping device served as power transmitter between the fascial thread and the load cell [7].

### 2.3. Development of the Implantable Micro-Sensor

#### 2.3.1. Specification Sheet

The following specifications were defined:

- measurement of suture tension “on the thread”;
- dimensions not exceeding 10 mm × 5 mm × 5 mm;
- implantable device in a coupled system with an implantable data logger;
- external connect board;
- data registration over a period of at least 24 h;
- suture tension measurement every second;
- measuring range 0–3 N, overload capacity of 20 N.

**Figure 2:**
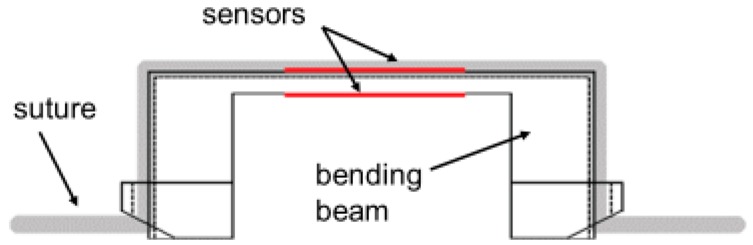
Schematic drawing of the sensor device.

**Figure 3:**
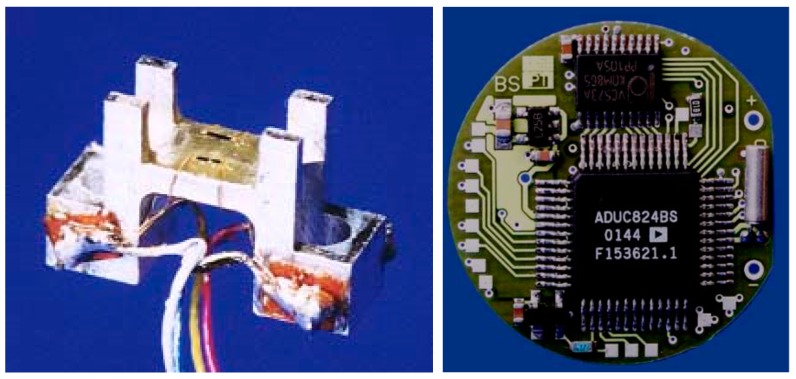
“Naked” implantable sensor device (6 mm × 3 mm × 1 mm) (**left**) and implantable data logger (Diameter 25 mm) (**right**).

These specifications were best met applying a bending beam construction (Figure 2 and Figure 3). All developmental work and the sensor realization were done together with the Fraunhofer Institute for Production Technology (Aachen, Germany) and have already been published elsewhere [6,8].

#### 2.3.2. Calibration and Accuracy

All sensors underwent an extensive offline qualification and calibration in a spring-load calibrator. First, 15 measurements were taken in each direction from 0 to 3 N (200% overload) and from 3 N to 0 N to perform the basic calibration. Hysteresis (the reproducibility of sensor values under application of a defined load) and the rheology (drift of measurement up to 20 min after a 100% overload was applied or removed) were checked. Sensors underwent a further five-point calibration with a standard load of 1 N immediately before implantation and a check for accuracy of measurement immediately after explantation.

#### 2.3.3. In Vivo Testing of ISDs

**Figure 4:**
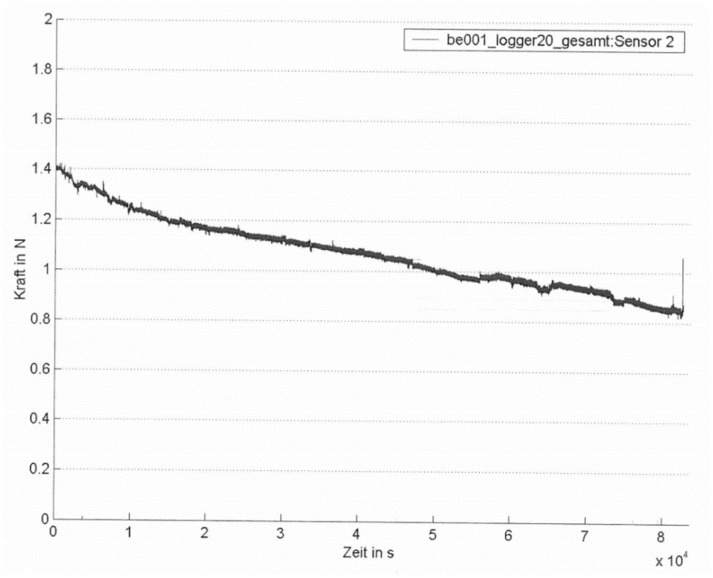
Example of an individual suture tension diagram after laparotomy closure without elevation of intra-abdominal pressure. Total recording time: 23.3 h (8.4 × 10^4^ s) (*x*-axis label: time in s; *y*-axis label: force in N).

**Figure 5:**
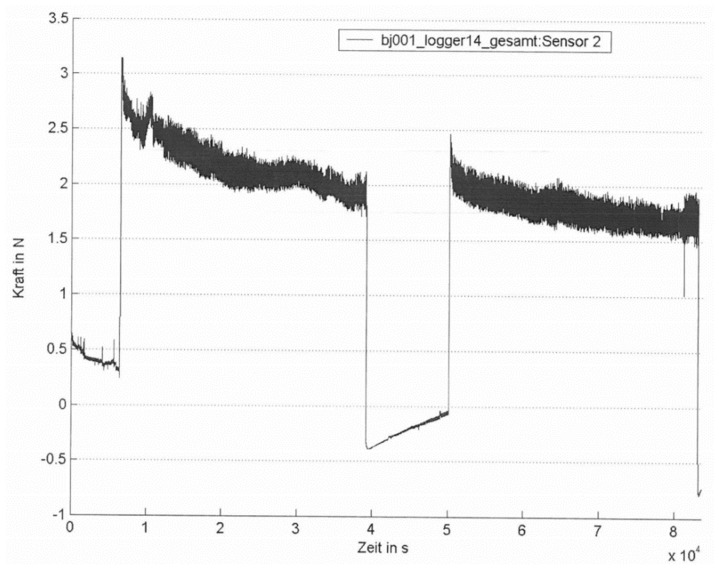
Example of an individual suture tension diagram (intervention group). Two 9 h intervals with 30 mm Hg IAH. Total recording time: 23.3 h (8.4 × 10^4^ s). (*x*-axis label: time in s; *y*-axis label: force in N).

In a feasibility study in pigs, the function of the ISDs and the long-term development of suture tension were evaluated. Midline laparotomies in 10 pigs with a body weight of approximately 50 kg were closed by one surgeon using size 1 continuous Vicryl sutures (Ethicon, Norderstedt, Germany) with a suture-length–wound-length ratio of 4:1. Two independent sensors were fitted on each suture line, one in the upper half and one in the lower half of the incision to allow for internal control. The initial suture tension given to the suture ensured close approximation of the incisional edges, but ruled out any tissue overlap or a bulging of the tissue between the stitches. Set off suture tension after completion of the suture was registered. Suture tension measurement and registration were then started with a frequency of 1/s. Six animals (hereafter called the “control group”) remained under general anesthesia during the entire experiments without any further intervention. In four pigs (further called “intervention group”), the setting was identical except for the application of two 9 h intervals with elevated intra-abdominal hypertension (IAH). IAH with a pressure level of 30 mm Hg was obtained and controlled by laparoscopic capnoperitoneum via a commercially available CO_2_—insufflator. The two intervals with IAH were interrupted by a 3 h interval with a depressurized abdominal cavity. After 24 h, the sensors were explanted, the loggers were read out, and the data were analyzed. All animals were sacrificed thereafter. Measurement values were depicted graphically (Figure 4 and Figure 5).

#### 2.3.4. Analysis of Measurement Values

**Figure 6:**
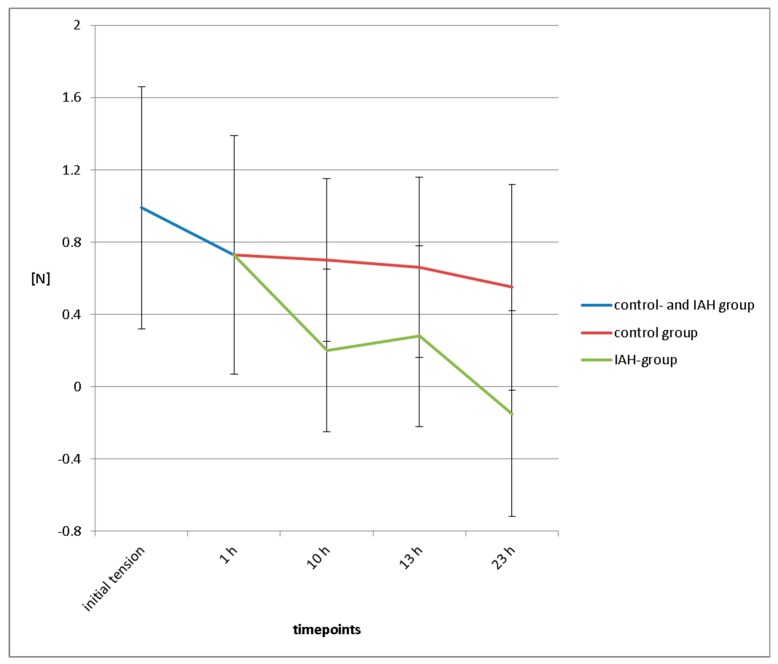
Mean suture tension levels immediately after completion of the suture and after 1, 10, 13, and 23 h in the control and intervention groups, depicted together with standard deviations.

Descriptive statistics with mean values and standard deviations of the study groups were calculated and depicted graphically (Figure 6). In this context, comparing the control group and the intervention group, we decided against further statistical analysis in order not to overstrain the validity of this feasibility study.

## 3. Results

### 3.1. Results of Suture Tension Reproducibility and Precision In Vitro (Knot-Trainer)

Between the different surgeons a suture tension variability of 235% was registered when the target suture tension was set to be “low,” of 69% when the target suture tension was set to “adequate,” and of 116% with the target set to “high.” The mean individual precision of suture tension varied between 407% (suture tension perceived as “adequate”), 242% (suture tension perceived as “low”), and 162% (suture tension perceived as “high”). Table 1 displays the results of target suture tension with mean values, interquartile ranges, and reproducibility/precision.

### 3.2. Results of Macro Sensor Experiment

The suture tension applied by the surgeon when suturing in running technique reached peak values between 10 and 20 N. After completion of the suture with the fascial edges adapted, the suture tension declined to values of 1 N within approximately 15 min.

### 3.3. Results of Experimental Evaluation of ISDs

#### 3.3.1. Sensor Accuracy

Contour accuracy and sensitivity during testing revealed the sensors suitable for force signals expected in laparotomy closure with a strain gauge expansion of 0.2% to a force signal of 1 N.

Sensors finally used in the experiment had a hysteresis and a rheological drift <0.05 N in the calibration process described. Five-point calibration with a standard load of 1 N immediately prior to implantation and a check for accuracy of measurement immediately after explantation confirmed this sensor accuracy in all sensors applied.

#### 3.3.2. Sensor Function

The sensors in eight animals registered suture tension dynamics reliably throughout the entire period. In two animals, the sensors malfunctioned due to solder breakage. These animals were excluded from further analysis. No problems with the data registration and transfer were detected between ISD, data loggers, and external connect boards. Following the elevation and normalization of intra-abdominal pressure in the intervention group, sensors directly and repeatedly registered the rise and fall of suture tension without delay.

#### 3.3.3. Suture Tension to Achieve Fascial Closure

Mean initial suture tension to achieve abdominal closure in pigs was 0.99 N ± 0.67 N (Figure 6).

#### 3.3.4. Suture Tension Dynamics

One hour after fascial closure, the mean suture tension had declined to 0.73 ± 0.66 N, a loss of 26% of initial tension. After 10 h, suture tension in the control group had fallen to 0.7 ± 0.65 N, and in the intervention group (after 9 h of abdominal hypertension) had fallen to 0.2 ± 0.45 N (−29% of initial suture tension in the control group and −80% in the intervention group). After 13 h, mean suture tension in the control group was 0.66 ± 0.56 N (−33% of initial suture tension), suture tension in the intervention group showed a recovery to 0.28 ± 0.5 N (+40% compared to suture tension directly after depressurization, −72% compared to initial suture tension). At the end of the experiment, mean suture tension in the control group was 0.55 ± 0.45 N (−45% of initial suture tension), and in the intervention group mean tension was −0.15 ± 0.57 N (Figure 6).

## 4. Discussion

### 4.1. Comparison of This Study to Prior Work in the Literature

In a series of animal experiments our scientific working group evaluated the influence of suture tension on laparotomy healing.

These studies analyzed the mechanical properties of laparotomies closed with different suture tension [9,10], the influence of suture tension on the ultrastructural composition of the healing incision (collagen fibril diameters, quantity and quality of collagen formation) [11,12], the influence of suture tension on tissue perfusion of the incisional region [13], and a novel technique to close laparotomies without direct sutures to the incisional edges [14].

The results of these studies substantiated the assumption that suture tension is a relevant influencing factor for fascial healing. The lack of adequate sensors measuring suture tension dynamics during suturing and after the suture has been accomplished has to be considered as a relevant impediment to scientific progress in this area of surgical research. Multiple publications have dealt with this problem.

In as early as 1989, Pollock et al. described inadequate suture tension as a risk factor for IH development [15]. The authors were able to predict future IH formation in laparotomies closed by excessive tension. Similar conclusions were drawn in studies by van Ramshorst et al. in 2010 [16] and Xing et al. in 2013 [17]. Searching the PubMed database using the keywords “suture tension measurement,” “force sensing in sutures,” “force sensing abdomen,” and “implantable sensors” resulted in 875 articles and reports. The description of research work leading to the development of an implantable sensor device allowing the registration of long-term suture tension dynamics in vivo as described in our article could not be found in the literature. 

Two recent articles focused on the development of sensors measuring suture tension in vitro [18] and in different types of tissue in vivo in rabbits [19]. While Horemann et al. [18] developed a force feedback tool to give the surgeon better control of the applied suture tension, Klink et al. [19] developed a bulky sensor over which single sutures were made in different tissues. The curve progression in these experiments was very similar to the curves registered in the control group in our experiment. Kiriyama et al. [20] presented results of sensor development and of cadaveric experiments in 2014. The sensors were also developed after previous FEM analysis and worked according to the same principles as ours. Measurement focused on suture tension in the knee joint. Suture tension under passive movement was up to 40-fold higher than the tension measured in our study after laparotomy closure. Kiriyama judged this excessive tension as a consequence of the ridged tissues in cadavers and as a clear limitation. In 2018, Roca et al. published the development of a forceps to measure the tension forces in the abdominal wall during laparotomy closure and the distances between wound edges. They applied the forceps in an experiment in pigs. This large, handheld transducer allows for the measurement of momentary forces and no long-term measurement [21]. In 2017, von Trotha et al. published a study using the knot-trainer designed by our working group with results very similar to those described in this article and drew the same conclusions [22].

ISDs are currently being developed in a rapidly growing field of research for medical applications in almost all medical specialties, measuring strain and stability in artificial joints, heart rate, blood pressure, blood glucose, oxygen saturation, bladder pressure, and many more. These sensors will increasingly find their way from experimental use into clinical application in the future [23].

### 4.2. The Myth of Controlling Suture Tension

When knotting sutures, the surgeon relies on the tactile and optical control of the thread and the haptic feedback he obtains from the tissue, the thread, and the knot before finally adjusting the suture tension he considers as “adequate” [24]. Surgeons’ faith in their ability to control suture tension is high, the argument most often given is a vague “feeling for what the tissue needs” and “surgical experience.” The experiments with the sensor-based “knot-trainer” demonstrating a wide intra- and inter-individual variability of suture tension and a low precision are suitable to shake surgeons’ belief in their ability to control suture tension.

In our experiment, surgeons achieved the lowest inter-individual variability of suture tension and the highest precision when they sutured with high suture tension, most probably due to a better tactile reception.

Additionally, it can be suspected that we have a subconscious feeling that tissue sutured together tightly will also heal firmly. We have clear evidence that the contrary is true.

**Figure 7:**
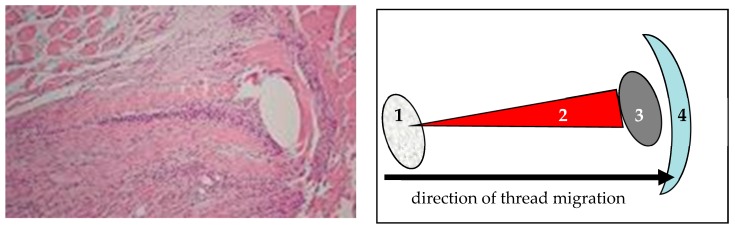
Comet’s tail phenomenon due to suture migrating through the abdominal wall in the direction of the incision [6,17]. (1) Original position of the thread; (2) comet’s tail consisting of tissue necrosis and inflammatory cells; (3) position of the thread at the time of cross section; (4) tissue necrosis due to compression.

In cross sections of the excised abdominal wall after closure of midline laparotomies, we found signs of sutures migrating from their original position laterally towards the edge of the incision, leaving behind tissue necrosis like a comet’s tail and compressing and damaging the tissue in front of the suture (Figure 7).

Our experimental studies evaluating the influence of suture tension provides us with valuable information about the effect of suture tension on the mechanical stability and ultrastructure of the healing laparotomy [9,10,11,12,13,14]:

- High suture tension leads to the formation of mechanically weaker scars than low suture tension.
- High suture tension has a negative influence on abdominal wall perfusion.
- Laparotomies closured with low suture show a significantly quicker transformation of mechanically instable collagen type III into mechanically stable collagen type I paralleled by the development of significantly larger collagen fibril diameters in the incisional region.
- The collagen content of the incisional region closed with high suture tension is significantly lower compared to laparotomies closed with low suture tension.
- Laparotomy closure in dogs without direct sutures to the incisional region leads to a mechanically stable healing of the incision if tearing forces are transferred by a “bridging” technique.

### 4.3. The Rationale for the Development and Use of ISDs Measuring Suture Tension Dynamics

The migration of the thread is to be considered as an “auto-regulation” of suture tension. If the tension given to the suture by the surgeon is too high and/or if a rise in intra-abdominal pressure leads to a distension of the abdominal wall, the suture cuts through the tissue and loses tension consecutively. Sutures cutting through until they reach the incision lose their retention in the sutured tissue and might lead to an acute failure of laparotomy closure with the development of a ruptured abdomen. If the migration stops anywhere before the incision is reached, the suture might lose tension to an extent that the incisional edges lose sufficient contact to allow for the formation of a mechanically stable scar [25].

### 4.4. Measuring Suture Tension in a Highly Dynamic Surrounding: A Genuine Challenge in Terms of Measurement Technology

Metrological requirements in medicine are special, the empirical data transfer from classical measurement technology is limited and conventional industrial sensors or transducers are often not applicable:

- Longer-term measurement of suture tension with sensors is hampered by the lack of coupling points and fixed points.
- Sutures in the abdominal wall include different tissues such as fascia, muscle, and fatty tissue. A material constant of these tissues does not exist, as biological tissues are inhomogeneous and anisotropic, and characteristics are highly time-variant.
- The extent of reversible elastic expansion and irreversible elongation of both tissue and thread cannot be estimated.
- The measuring range is small, and its variability high. The dimension of ISDs is important, and bio-compatibility plays a role in implantable devices.
- In a dynamic setting such as the abdominal wall, sensors have to be positioned precisely on site from which the data originates.

### 4.5. Sensor Development, Function, and Results of Experimental Use

The development of an ISD device measuring long-term suture tension dynamics in the abdominal wall in vivo was successful. This micro-sensor allows for the online registration of long-term suture tension development in a highly dynamic surrounding under challenging conditions for measurement technology. The transfer of technical requirements and measuring range identified using the macro sensor was shown to be possible. With the help of these sensors, it becomes clearer for the first time what happens to suture tension in the abdominal wall in vivo after the suture has been accomplished. The repetitive quick response of the sensors to the rise and fall of intra-abdominal pressure proved the functionality of the system. As solder breakage was the only reason for sensor dysfunction in our experiment, a more solid encapsulation of the sensor in the region of the solder joints has to be ensured prior to further experiments.

Virtually nothing is known about what the fascia needs in terms of suture tension or which suture tension is detrimental for fascial healing. This situation is worsened by the fact that, although surgeons have the opinion to be able to control and reproduce suture tension levels, the result of our experiment with the knot-trainer proved the opposite. Target suture tension of mock fascial closure considered as “adequate” for fascial closure was in fact more than 2.6-fold higher than the mean initial suture tension measured directly after fascial closure in the experiment with the ISD device. Inadequately high suture tension, described by Franz et al. [25] and demonstrated in our cross sections of sutured abdominal walls, leads to tissue strangulation, necrosis, inflammation, the liberation of lytic enzymes, and tissue destruction. If the thread cutting through the abdominal wall in the direction of the incision is the expression of an auto-adjustment of suture tension to reach a tissue-specific suture tension optimum, an uncontrolled auto-adjustment puts laparotomies at risk of acute or chronic failure. It is interesting that mean suture tension considered as “low” during mock fascial closure was in the range measured for fascial closure in the in vivo experiment (0.9 N vs. 0.99 N). As suture tension felt to be “adequate” might in reality be too high, the subconscious fear that “low” suture tension might just be too low to allow undisturbed healing drives us to apply higher suture tension than is really beneficial for fascial healing. How difficult it is to control suture tension and how variable the suture tension values applied for fascial closure are can be derived from the high standard deviation of initial suture tension registered in our experiment with the ISD. All sutures were performed by the same surgeon, suturing with the lowest possible suture tension to achieve fascial closure. Still, standard deviation was 0.67 N, above and below the mean suture tension of 0.99 N.

Though the results of our experiments were expected, the reality is still surprising. 

A continuous suture for laparotomy loses almost 50% of its initial tension after 23 h, with a steep loss of 26% as soon as 1 h after laparotomy closure, even if the animal remains under constant anesthesia, ruling out relevant changes in intra-abdominal pressure due to spontaneous or reflectory muscle activity. Only the minority of our patients remain under narcosis postoperatively; in the majority, narcosis is terminated directly after the end of the operation. The consequences are an elevation of intra-abdominal pressure through rising muscle tone, active movements, coughing, vomiting, defecating, laughing, etc. Fifteen to 20% of patients after laparotomy develop an unrecognized elevated intra-abdominal pressure. About one-third of patients admitted to intensive care wards have an elevated intra-abdominal pressure or an abdominal compartment syndrome [26,27]. The intra-abdominal pressure of 30 mm Hg applied in the intervention group was high (grade IV IAH), and the setting of two 9 h intervals with this elevated pressure unusual—but this design served two aims: to find out how the sensors react to a steep rise and fall of suture tension and to clarify a possible recovery of suture tension after depressurizing of the abdomen.

Already after the first interval with IAH, the mean suture tension decreased by 80% compared to the mean initial tension. A recovery of mean suture tension of 8% compared to the initial mean tension was registered. This was a rise of mean tension from 0.2 to 0.28 N in absolute numbers. It can only be speculated if this rise of suture tension was caused by the elasticity of the abdominal wall and/or the thread or was due to tissue edema in the sutured tissue following tissue damage by the strangulation of the sutured tissue. After the second interval with IAH negative mean values of suture tension were registered. We interpret these values to result from a complete loss of suture tension with dislocation of the sensor and interference with the adjacent tissue.

The study by Klink et al. [19] is the only study published so far with a design similar to ours, except that no implantable sensors were used and registration of suture tension was made for only 60 min. Klink et al. also described a correlation between the collagen content of the sutured tissue and the loss of suture tension. The higher the collagen content, the lower the loss of suture tension. The rapid initial loss of suture tension was interpreted by the authors as an expression of sutures cutting through the tissue, the slower decrease following the initial phase as plastic deformation of the sutured tissue.

What are the consequences of these results?

If we were able to define a tissue-specific suture tension optimum for laparotomy closure with further research, surgeons might be able to be trained to suture with this tension by the use of “intelligent threads” [28] or simpler by using a spring balance to achieve the optimal suture tension. To our mind, this aim cannot be reached for the following reasons:

- The abdominal cavity and the abdominal wall are too dynamic and anisotropic and the influencing factors on compliance, elasticity and structure are too variable to allow standardization.
- Surgeons will not accept the need as they have not really accepted the need for change in this field during the last 70 years.

As surgeons have tried in vain to stabilize the abdominal wall as a dynamic structure by “static” sutures that damage the sutured tissue by cutting through, would “dynamic” sutures with a much higher elasticity than the sutures used nowadays be a solution? The producers of suture materials promote materials for fascial closure to be elastic, but this elasticity is too limited and the force to stretch these sutures is much higher than it would have to be in knowledge of the forces working in the incisional region. Closing the fascia by a rubber band could be worth trying but should be combined with measures to prevent interposition of omental fat or a bowel loop.

Abdominal incisions are closed with direct sutures in the incisional edges, and there have been only slight technical variations in this process over the last 100 years. To leave the beaten path of laparotomy closure, we have proved in dogs in an experimental study that mechanically stable laparotomy closure is feasible without directly suturing the fascia. In a “bridging technique”, the tearing forces working on the incision have been transferred from one side of the incision to the other resulting in high mechanical stability, an undisturbed tissue perfusion and an advantageous ultrastructural composition [14]. This technique comes closest to studies evaluating the effect of prophylactic mesh re-enforcement in laparotomies at risk of failure, studies that demonstrate a relevant reduction in IH incidence [29]. It might only be difficult to identify patients with “laparotomies at risk.” They are of course those with aortic aneurysms and rare collagen disorders. However, with the known and well investigated risk factors, obesity, diabetes, smoking, re-operation, etc. and the prevalence of these risk factors in our population—will not everyone qualify for a prophylactic mesh in face of an IH-incidence of about 20%?

## 5. Conclusions

- Long-term suture tension dynamics can be measured in vivo by the implantable sensor device developed under normal and elevated intra-abdominal pressure levels.
- Surgeons are unable to reproduce target suture tension by visual and tactile control only and tend to apply high suture tension, which has negative effects on wound healing.
- In face of 50% loss of initial suture tension in laparotomies under normal intra-abdominal tension after 23 h and a near total loss of initial suture tension after intermittent elevation of intra-abdominal pressure, alternative methods of laparotomy closure must be considered to reduce IH incidence.

## Figures and Tables

**Table 1:** Mean suture tension, reproducibility, and precision during mock fascial closure experiment using the knot-trainer.

Target Suture Tension for Fascial Closure	Mean Suture Tension and SD [N]	Interquartile Range 25/75 [N]	Inter-Individual Variability of Mean Values	Intra-Individual Variability during Repeated Knotting
“low”	0.9 ± 0.47	0.56–1.43	235%	242%
“adequate”	2.64 ± 0.89	1.58–3.39	69%	407%
“high”	4.18 ± 1.64	2.86–6.21	116%	162%
